# Temporary uterine artery blocking and uterine artery embolization in treating cesarean scar pregnancy

**DOI:** 10.20452/wiitm.2024.17890

**Published:** 2024-07-31

**Authors:** Kefei Zeng, Xianghua Lei, Tingting Xia

**Affiliations:** Department of Obstetrics and Gynecology, Affiliated Hospital of Jinggangshan University, Ji’an, Jiangxi Province, China; Department of Reproductive Medicine, Affiliated Hospital of Jinggangshan University, Ji’an, Jiangxi Province, China

**Keywords:** cesarean scar pregnancy, cesarean section, temporary uterine artery blocking, uterine artery embolization

## Abstract

**INTRODUCTION:**

Cesarean scar pregnancy (CSP) is a rare form of ectopic pregnancy. Lack of timely CSP treatment can lead to severe postpartum bleeding, affect fertility, and threaten patients’ life and health.

**AIM:**

This work explored the use of laparoscopic temporary uterine artery blocking (TUAB) and uterine artery embolization (UAE) in treating CSP.

**MATERIALS AND METHODS:**

For the purpose of the study, 60 patients with CSP were selected and equally divided into the UAE group and the TUAB group. Both groups underwent cesarean scar lesion repair (CSLR) after the procedure. The differences in surgical outcomes, β‑human chorionic gonadotropin (β‑HCG) levels, ovarian reserve, endocrine function indicators, as well as the incidence of complications were compared between the groups.

**RESULT:**

The TUAB group presented a shorter length of hospital stay, shorter vaginal bleeding time, shorter menstrual recovery time, and shorter mass disappearance time than the UAE group (all P <0.05). The patients in the TUAB group had lower β‑HCG, estradiol, and progesterone levels, and higher levels of luteinizing hormone and follicle‑stimulating hormone (all P <0.05). Furthermore, the TUAB patients had a larger mean ovary diameter, an increased antral follicle count, and an elevated level of anti‑Müllerian hormone, as compared with the individuals treated with UAE (all P <0.05). The total incidence of complications in the UAE and TUAB groups was 23.33% (7/30) and 6.67% (2/30), respectively (P <0.05).

**CONCLUSIONS:**

Laparoscopic TUAB for CSLR proved to be a more effective and safer CSP treatment method than UAE.

## INTRODUCTION 

Cesarean scar pregnancy (CSP) refers to an ectopic pregnancy in which the fertilized egg implants in the scar of a previous cesarean section incision.^1^^;^^2^ The increasing rate of cesarean sections has led to a corresponding rise in the incidence of CSPs in the recent years. If CSP is not diagnosed and treated promptly, it can cause severe postpartum bleeding and lead to hysterectomy.^3^ Moreover, CSP can affect patients’ fertility and pose a threat to their health and lives.^4^ In clinical practice, CSP is commonly treated with local or systemic drugs. Although conservative drug treatment offers some therapeutic effects, miscarriage is not complete and scraping is still required. In severe cases, CSP can cause massive bleeding, which may compromise patient outcomes and increase the risk of morbidity and mortality.^5^^;^^6^

Uterine artery embolization (UAE) is a novel and minimally invasive technique that has been widely used in the treatment of postpartum hemorrhage, adenomyosis, uterine fibroids, and other diseases.^7^ In addition, UAE has been used with great success in the treatment of CSP after caesarean section.^8^^;^^9^ Temporary uterine artery blocking (TUAB) is a procedure consisting in occlusion of uterine arteries during surgery using either electrocoagulation or suture ligation to reduce intraoperative bleeding.^10^ TUAB can also be used in the treatment of CSP after a caesarean section, but the reports on this procedure are scarce. ^11^

This study compared the clinical efficacy of UAE and TUAB in treating CSP, and analyzed the differences in ovarian reserve function, ovarian endocrine function, and incidence of complications between these surgical interventions. It was intended to give a reference for the selection of clinical treatment methods for CSP, and to improve patient prognosis and survival.

## AIM 

The aim of the study was to analyze the efficacy of TUAB and UAE in treating CSP.

## MATERIALS AND METHODS 

### General data 

For the purposes of the study, 60 pregnant women with CSP admitted to the Affiliated Hospital of Jinggangshan University between February 2022 and February 2023 were selected and assigned to the UAE group (30 patients) and the TUAB group (30 patients) according to the order of admission. The mean (SD) age of the patients in the UAE group was 28.09 (2.13) years (range, 23–35 years), and their mean (SD) weight was 63.14 (4.83) kg (range, 55–80 kg). According to the position and depth of embryo implantation, CSP can be classified into 3 types: type I (superficial type), type II (invasive type), and type III (complete invasive type). The UAE group included 9 cases of type I CSP, 10 cases of type II CSP, and 11 cases of type III CSP. The patients had between 1 and 3 previous caesarean sections (mean [SD], 1.59 [0.42]), whereas the mean (SD) time from the last caesarean section to the present CSP was 3.71 (2.35) years. The mean (SD) duration of amenorrhea among the UAE patients was 45.63 (8.97) days (range, 25–73 days), and their mean (SD) mass diameter was 2.8 (0.68) cm.

The patients in the TUAB group were at a mean (SD) age of 28.57 (2.44) years (range, 25–39 years) and their mean (SD) weight was 62.57 (4.91) kg (range, 57–78 kg). This group included 10 cases of type I CSP, 11 cases of type II CSP, and 9 cases of type III CSP. The patients had between 1 and 3 previous caesarean sections (mean [SD], 1.6 [0.28]), and the mean (SD) time from the last caesarean section to this pregnancy was 3.63 (2.5) years. The mean (SD) duration of amenorrhea in the TUAB group was 48.52 [9.14] days (range, 25–75 days) and the mean (SD) mass diameter was 2.91 (0.7) cm. No significant differences between the groups were observed with regard to general characteristics.

The patients enrolled in this study had to meet the following criteria: 1) a history of caesarean section; 2) ultrasound or other imaging examinations showing CSP with an empty uterine cavity and cervical canal, thinning or absence of the myometrium layer between the gestational sac and bladder, and low resistance blood flow signals in the nourishing layer of the scar; 3) complete β-human chorionic gonadotropin (β-HCG) and imaging data; and 4) indications for UAE and TUAB. The patients were excluded if they had any of the following conditions: 1) coagulation abnormalities; 2) contraindications to medication or surgery; 3) malignant tumors; 4) a history of preterm labor, recurrent miscarriage, or fetal malformation; 5) concomitant gynecological diseases, such as uterine fibroids, adenomyosis, or ovarian cysts requiring simultaneous surgical treatment; and 6) previous treatment for CSP.

### Ethics approval and consent to participate 

Informed consent was obtained from all participants and / or their legal guardian(s). This included information regarding informed consent obtained from a parent or legal guardian of any participant below the age of consent. Adherence to the guidelines outlined in the Declaration of Helsinki was confirmed. The study was approved by the medical ethics committee of the Affiliated Hospital of Jinggangshan University (AHJU035).

### Treatment methods 

All patients underwent complete blood count, urinalysis, electrocardiogram, and other tests after admission. The patients included in the UAE group underwent UAE treatment followed by laparoscopic cesarean scar lesion repair (CSLR). The patients in the TUAB group were subjected to laparoscopic TUAB treatment followed by CSLR. Both groups underwent CSLR 1 to 3 weeks after their respective UAE or TUAB procedures.

In the UAE group, the patient was placed in a supine position, a sterilized cloth was placed to isolate the site of surgical incision, and local anesthesia was administered through the right femoral artery using the Seldinger technique. Then, a 5F vascular sheath was inserted into the uterine artery for selective catheterization. Subsequently, 100 ml of iodinated contrast agent was injected for uterine artery angiography. Once the catheter entered the uterine artery, the morphology of the bilateral uterine arteries and the change in uterine volume were observed. After confirming lesion location, 100 ml of physiologic saline containing methotrexate was infused into the uterine artery vein bilaterally, followed by embolization with 80 gelatin sponge particles. Angiography was performed to evaluate the imaging status of the uterine arterial trunk, branches, and terminal vessels, and to assess the embolization effect. The catheter was then removed and pressure was continuously applied for 10 minutes to avoid bleeding.

In the TUAB group, the patient was placed in a supine position and administered general anesthesia after tracheal intubation. The ureteral course was observed under laparoscopy and the uterine artery was fully mobilized by cutting the broad ligament posterior leaf 2 cm above the lateral part of the sacrospinous ligament. Arterial blood flow was blocked by ligating the uterine artery with a 10–0 silk suture. Subsequently, 100 ml of physiological saline containing 6 U of vasopressin was injected at the junction of the uterine scar lesion and the uterine body, and the bladder was pushed down to the cervical ostium. Suction curettage was performed under laparoscopy, and the scar was reinforced and sutured with absorbable thread after the procedure. The surgical treatment was considered satisfactory when there was no residual embryonic tissue in the uterine cavity, no scar diverticulum, and the suture line did not penetrate the uterine cavity.

**Figure 1:**
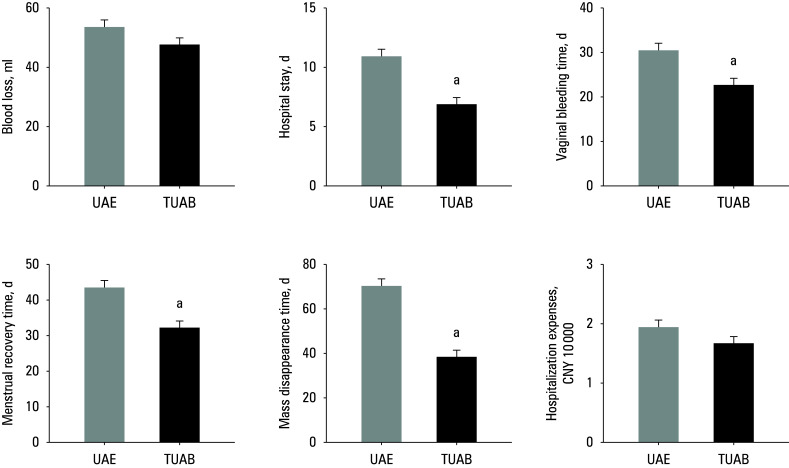
Indicators of surgical outcomes

**a** P <0.05

Abbreviations: CNY, Chinese yuan renminbi; TUAB, temporary uterine artery blocking; UAE, uterine artery embolization

The UAE and TUAB groups underwent CSLR via UAE treatment and laparoscopic TUAB treatment, respectively. The procedural steps for both groups were identical and performed using the same laparoscope. CSLR was performed as follows: a purplish-brown mass was observed at the level of the internal inlet of the cervix in the external side, and the bladder peritoneum was observed to be folded and purplish-brown on the inner side. After opening the bladder peritoneum, the uterine myometrium at the caesarean section scar site was found to be thinner, most of the muscle fibers were ruptured, and the uterus itself was almost ruptured as well. The pregnancy tissue and blood clots were carefully separated and removed, and the pregnancy tissue tightly adhering to the uterus was excised. The original caesarean section incision was fully opened, the edges were trimmed, and the incision was sutured using an absorbable thread, leaving no cavity. All procedures were performed by a single surgeon with extensive experience in laparoscopic surgery, which allowed for maintaining consistency with respect to surgical habits.

### Observation indicators 

First, intraoperative blood loss (IBL), length of hospital stay (LOS), vaginal bleeding time (VBT), menstrual recovery time (MRT), and mass disappearance time (MDT) were recorded. Then, before and 1 to 2 days after CSLR, fasting morning venous blood samples (approximately 3 ml) were collected. The chemiluminescent immunoassay was used to detect serum levels of β-HCG, and an automatic biochemical analyzer was employed to measure luteinizing hormone (LH), follicle-stimulating hormone (FSH), estradiol (E2), and progesterone (P4) levels. Subsequently, before and after treatment, ultrasonography and other techniques were used to examine the mean ovary diameter (MOD), anti-Müllerian hormone (AMH) level, and the antral follicle count (AFC). Finally, the incidence of complications, such as abdominal pain, intrauterine adhesions, and premature ovarian failure was recorded.

### Statistical analysis 

Statistical analysis was performed using SPSS 22.0 (IBM Corp., Armonk, New York, United States). Continuous variables are presented as mean (SD) and were compared using the t test. Categorical variables are presented as frequency (percentage) and were compared using the χ2 test. A P value below 0.05 was considered significant.

## RESULTS 

### Surgical outcomes 

Differences in IBL, LOS, VBT, MRT, MDT, and hospitalization expenses between the UAE group and the TUAB group are shown inFigure 1A–F. There was no significant difference in IBL and hospitalization costs between the patients receiving UAE and TUAB. However, in the TUAB group, LOS, VBT, MRT, and MDT were significantly shorter than in the UAE group.

**Figure 2:**
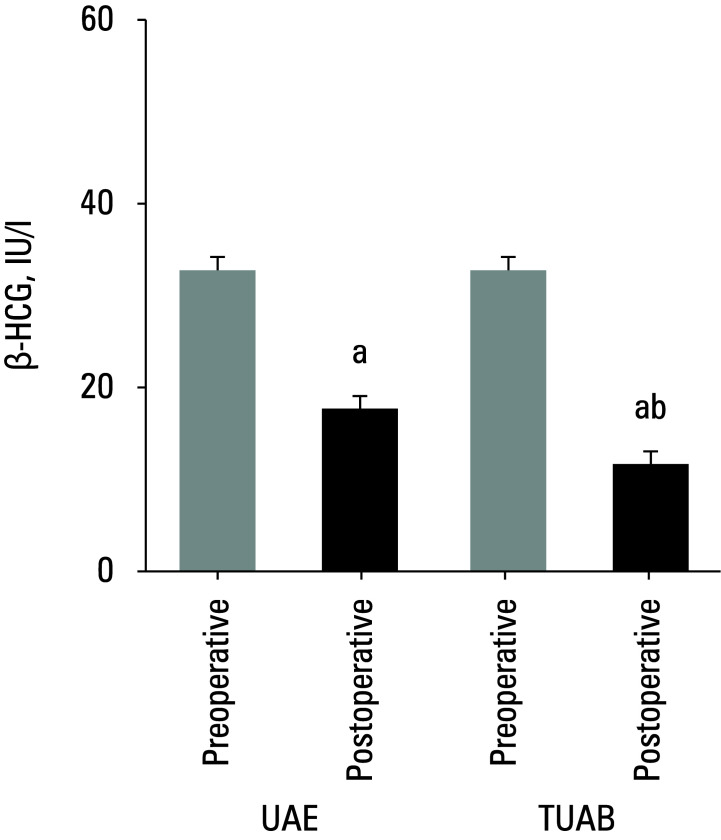
Comparison of pre‑ and postoperative β ‑human chorionic gonadotropin (β ‑HCG) levels in patients treated with different surgical approaches

**a** P <0.05 vs preoperative level

**b** P <0.05 vs the UAE group

Abbreviations: seeFigure 1

### β‑Human chorionic gonadotropin levels 

A comparison of β-HCG levels in peripheral blood of the UAE and TUAB patients is illustrated inFigure 2. No remarkable difference between the groups was observed before surgery and before CSLR. However, β-HCG levels in both groups of patients decreased after their respective treatments, showing a clear difference (P <0.05). Furthermore, the patients treated via TUAB showed a notably greater decrease in β-HCG levels after the treatment, as compared with the UAE group (P <0.05).

### Ovarian reserve function 

Differences in ovarian reserve function between patients treated with different approaches are presented inFigure 3. No visible difference was observed in MOD, AMH, and AFC values of the patients before their treatments. The post-treatment values of MOD, AMH, and AFC in both UAE and TUAB groups increased, showing a significant difference. Additionally, the post-treatment increase in the values of MOD, AMH, and AFC in the TUAB group was more pronounced than in the UAE group (P <0.05).

### Ovarian endocrine function 

The ovarian endocrine function of patients in the 2 groups was compared in terms of LH, FSH, E2, and P4 levels, as illustrated inFigure 4. No visible differences in hormone levels were found between both groups before surgery. The post-treatment LH and FSH levels were increased, while E2 and P4 levels were lower, showing visible differences when compared with the preoperative levels (P <0.05). In addition, the TUAB patients showed a greater increase in LH and FSH levels and a more pronounced decrease in E2 and P4 levels after treatment, as compared with the UAE group (P <0.05).

### Incidence of complications 

The incidences of abdominal pain, intrauterine adhesion, premature ovarian failure, amenorrhea, decreased menstrual volume, and uterine arteriovenous fistula in the patients after treatment were comparedFigure 5. In the UAE group, there were 2 cases (6.67%) of abdominal pain, 2 cases (6.67%) of intrauterine adhesion, 1 case (3.33%) of premature ovarian failure, 0 cases of amenorrhea, 2 cases (6.67%) of decreased menstrual volume, and 0 cases of uterine arteriovenous fistula. In the TUAB group, there was 1 case (3.33%) of abdominal pain, 2 cases (6.67%) of intrauterine adhesion, 0 cases of premature ovarian failure, 0 cases of amenorrhea, 0 cases of decreased menstrual volume, and 1 case (3.33%) of uterine arteriovenous fistula. The total incidence of complications (TIOC) was 23.33% (7/30) in the UAE group and 6.67% (2/30) in the TUAB group. Thus, as compared with the UAE group, the TIOC was lower among the patients subjected to the TUAB treatment (P <0.05).

**Figure 3:**
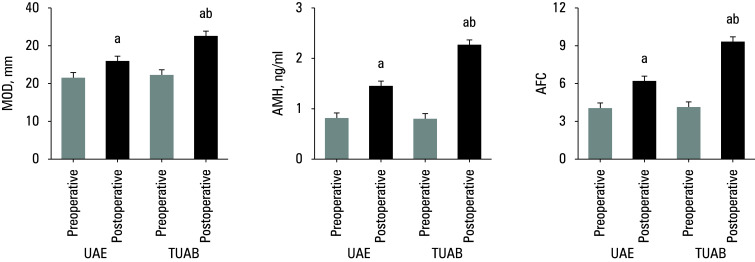
Comparison of ovarian reserve function indicators; **A** – mean ovary diameter (MOD); **B** – anti‑Müllerian hormone (AMH) level; **C **– antral follicle count (AFC).

**a **P <0.05 vs preoperative values

**b** P <0.05 vs the UAE group

Abbreviations: seeFigure 1

**Figure 4:**
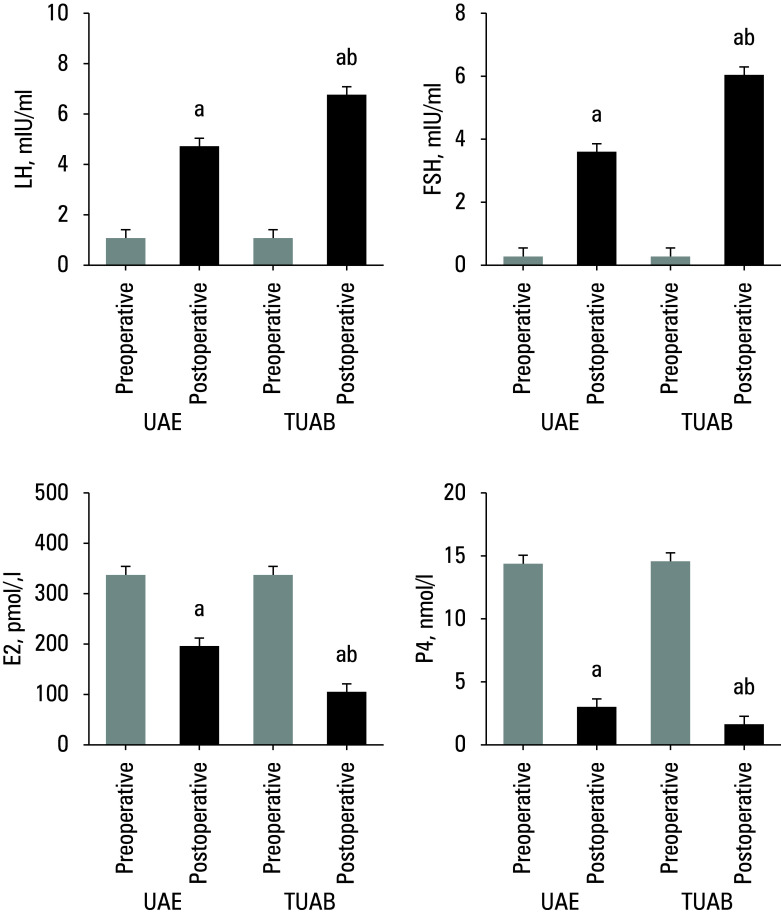
Comparison of ovarian endocrine function indicators; **A** – luteinizing hormone (LH), **B** – follicle‑stimulating hormone (FSH), **C** – estradiol (E2); **D** – progesterone (P4)

**a **P <0.05 vs preoperative level

**b** P <0.05 vs the UAE group

Abbreviations: seeFigure 1

**Figure 5:**
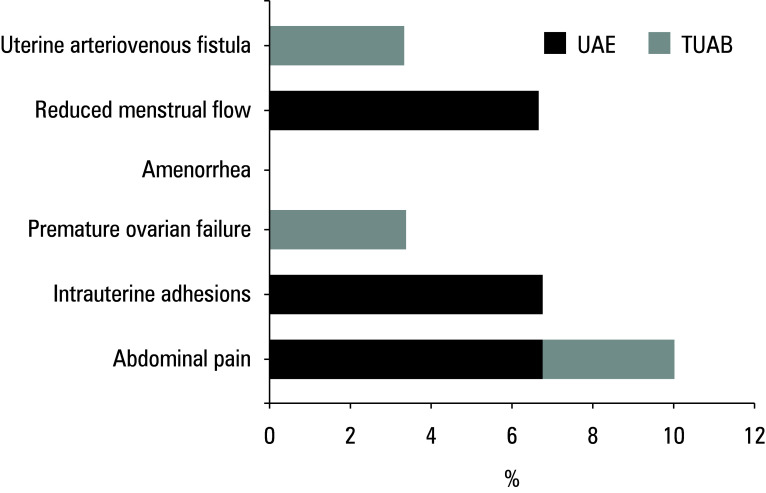
Incidence of postoperative complications

Abbreviations: seeFigure 1

## DISCUSSION 

CSP refers to the implantation of a pregnancy sac, fertilized egg, or embryo in a previous cesarean section scar.^12^ CSP is a long-term complication of a cesarean section, but its pathogenesis remains unclear. Since CSP has no obvious symptoms, patients often present with irregular vaginal bleeding or abdominal pain after amenorrhea.^13^ As CSP progresses, the risk of uterine rupture, placenta accreta, and bleeding increases significantly.^14^ Since there are individual differences in the thickness of the uterine muscle layer, blood β-HCG levels, and scar blood flow at the CSP scar site, there is no unified standard for the treatment of CSP.^15^ In clinical practice, open surgery, laparoscopic surgery, and hysteroscopic surgery are commonly used to treat the condition. Methotrexate can also be used locally or systemically.^16^^;^^17^ Methotrexate combined with curettage can ensure thoroughness of treatment, but it may also cause severe bleeding and even lead to hysterectomy, resulting in the loss of fertility and prolonged LOS.^18^ UAE is a minimally invasive treatment that involves direct occlusion of the uterine artery through a catheter, reducing the risk of postcurettage bleeding.^19^ This study evaluated the clinical effects of UAE and TUAB in treating CSP, and found that TUAB significantly shortened patient LOS, VBT, MRT, and MDT, as compared with UAE. Laparoscopic surgery allows for effective removal of residual embryonic tissue and evaluation of the suture effect until the scar diverticulum disappears, which improves the effects of scar repair surgery.^20^^;^^21^

UAE is a safe and effective method for CSP patients with elevated blood β-HCG levels and rich blood vessels.^22^ This study found that both UAE and TUAB treatments significantly reduced blood β-HCG levels, with a more significant reduction observed in the TUAB group. LH is a glycoprotein gonadotropin secreted by pituitary cells. A decrease in LH levels indicates poor ovarian function.^23^ FSH is a glycoprotein hormone synthesized and secreted by the pituitary gland that regulates ovarian follicle development and maturation.^24^ LH and FSH work together to promote ovarian maturation. E2 is primarily secreted by the ovaries and promotes endometrial growth in the proliferative phase. It is an important hormonal indicator of ovarian function.^25^ A decrease or decline in ovarian function can lead to abnormal increases in E2 levels.^26^ P4 is mainly secreted by the ovaries and corpus luteum and is a hormone that maintains normal reproductive function. It can be an inficator of ovarian function and fertility.^27^ Our results showed that after UAE and TUAB treatments, blood LH and FSH levels increased, while E2 and P4 levels dropped. The patients treated with TUAB exhibited a more significant increase in blood LH and FSH levels and a more marked decline in E2 and P4 levels, indicating that TUAB can better maintain the ovarian function and fertility in CSP patients.

Ovarian reserve refers to the primordial follicles contained within the ovarian cortex. In the state of reduced ovarian reserve function, the baseline FSH level increases and the AFC significantly decreases.^28^ MOD, AMH, and AFC are important predictors of ovarian reserve function. MOD refers to the mean value of the maximum diameters in 2 perpendicular planes of either ovary, with 20 mm adopted as the critical value. ^29^ AMH is a member of the transforming growth factor β superfamily, mainly secreted by granulosa cells of preantral and small antral follicles with a diameter of 4 mm, and it is a regulator of follicular growth and development.^30^ AFC refers to the number of follicles on both ovaries on days 2–4 of the menstrual cycle. This study indicated that after UAE and TUAB treatments, the values of MOD, AMH, and AFC increased, and the increase was more obvious in the TUAB group. AMH directly or briefly regulates follicular development and inhibits follicular growth to prevent rapid depletion of follicles.^31^ AMH level dynamics can predict changes in ovarian reserve early and accurately. AFC is an independent predictor and the best indicator of low ovarian response.^32^ Early AFC positively correlates with HCG levels.^33^

This study also suggested that the incidence of abdominal pain, uterine adhesions, premature ovarian failure, amenorrhea, reduced menstrual flow, and uterine arteriovenous fistula after TUAB treatment was significantly lower than after UAE treatment, indicating that TUAB might be more advantageous in reducing these potential adverse outcomes and complications often associated with postoperative wound infections. Some studies have proposed that prophylactic wound treatment can effectively reduce the incidence of surgical site infections and wound complications.^34^ However, this study focused on CSLR, whose application effect has not been specifically analyzed and requires further investigation. Therefore, TUAB treatment for CSP appears to better preserve ovarian reserve function in patients and reduce the incidence of surgical complications, thereby enhancing clinical treatment outcomes.

## CONCLUSIONS 

The use of laparoscopic TUAB for CSP treatment can shorten LOS, preserve the ovarian and uterine function, meet fertility needs, promote rapid postoperative recovery, and reduce the incidence of surgical complications. However, this study only evaluated the short-term efficacy of UAE and laparoscopic TUAB in treating CSP. More data are needed to assess the long-term safety of pregnancy. In summary, this work provided a reference for the selection of treatment options for CSP, such as laparoscopic TUAB and UAE.
